# Statutory or Voluntary

**Published:** 1956-04

**Authors:** A. V. Neale


### *Correspondence*

DEAR SIR,

*Statutory or Voluntary*

The observations of the Chancellor of the Exchequer given in the recent budget speech indicate once again that ways and means have got to be found to produce a real economy in public current expenditure. Enormous programmes of building have been sapping our national finances. The serious and backward structural conditions of very many of the hospitals taken over under the National Health Act in 1948, and especially the hospitals for the mentally ill, will and must continue to absorb a large percentage of the Minister of Health's available money for this the "only sacrosanct item" noted in the proposed permissible building programmes.

It is alarming to see it stated that the recent expansion of local authorities' expenditure "has been the worst feature of public finance this year". No doubt included in this is a certain amount of expenditure incurred in the administration of the ever-increasing statutory services which swell the demand for more and more official facilities and staff for their implementation. It would be out of place here to name and to discuss each of these, but there would appear to be in all this some scope for a careful reconsideration of why and how, in certain respects, some first-class and well-established voluntary services which have proved their great worth over the years, and especially so in the last ten years, are allowed to thin out or even to go into desuetude because "the need is now to be met by an administratively costly statutory system".

Fortunately, the peculiar tenacity of the voluntary spirit is not easily submerged. There are a great number of people in this country who, even now, are willing and able to give assistance on a voluntary basis. The real problem often lies in the provision for even minimal facilities and personnel for administrative and secretarial purposes in such voluntary organizations. Happily in some places, including Bristol, some highly valued assistance of this sort is forthcoming, but the method could be extended much more widely as a basis of setting voluntary services on a more steady and stable background. This is specially so in the voluntary assistance concerned with the many and varied social services, whose action in many ways could engender a strong liaison, say, with the statutory medical services. Through such channels, ways can be developed for educating the public in the availabilities and proper use of the Health Services. Liaison as between family doctors, the social services, and preventive medicine services, can quite appropriately be built up. The voluntary services can often act in a special way and to function as catalysers of activity on their own and as between groups of others. Child Health Services have and should, in fact, benefit greatly by these voluntary activities.

These fields of voluntary work might well, with greater general appreciation of their real worth and with their wider development, take again their full and proper place in our national organization, and be by no means a small factor in bringing back some of our former financial stability. Statutory organizations, whether for preventive or curative medical work, or for social and some educational purposes, have here and there tended to engulf the fire of the voluntary effort.

It is of special interest to note the remarks made by Dr. Charles White in his recent presidential address to the Society of Medical Officers of Health:

> "In discussing the advances that the M.O.H. might make into new territory with the aid of allies, Dr. White numbered among those allies the voluntary societies. In one respect these had a great advantage over official bodies: they could take action in individual cases, so far as their funds permit, without committing themselves to similar action in all the similar cases that might present themselves; and this made it easier for them to do pioneer work on a small scale. Many people were still anxious to give personal service to those in misfortune or distress, but on the other hand it was nowadays far harder to collect money for charitable purposes. 'While obviously no voluntary organization can be given a free hand to spend public money, I suggest that it would be well worth while widening the range of activities in which voluntary societies may be subsidized by local authorities'."

It seems, therefore, that a real search into the fields of voluntary effort, and in increasingly sympathetic and understanding approach to the minimal but basic financial needs to keep the work moving, without the recurrently alarming and sometimes despondent situation of worrying about how the office clerk or the administrative secretary's salary can be met, might be now very well worth while. Economy by and with the full use of voluntary services should be a rising force. The Treasury and the Local Authorities should now regard all this as a very important matter. They should seek the avenues whereby, either by full co-operation with or by more delegation of responsibility to voluntary societies, money can be saved.

Yours faithfully,

A. V. NEALE.

